# Temperature Dependence of Electrophoretic Mobility and Hydrodynamic Radius of Microgels of Poly(*N*-isopropylacrylamide)

**DOI:** 10.3390/gels4020037

**Published:** 2018-04-20

**Authors:** Yasuyuki Maki, Kentaro Sugawara, Daisuke Nagai

**Affiliations:** 1 Department of Chemistry, Graduate School of Science, Kyushu University, Fukuoka 819-0395, Japan; 2 Division of Molecular Science, Faculty of Science and Technology, Gunma University, Kiryu 376-8515, Japan; t14801041@gunma-u.ac.jp (K.S.); daisukenagai@gunma-u.ac.jp (D.N.)

**Keywords:** microgel, electrophoresis, light scattering

## Abstract

Electrostatic interactions in charged microgels, which are dominated by the microgel net charge, play a crucial role in colloidal stabilization and loading of small, charged molecules. In this study, the temperature dependences of electrophoretic mobility *μ* and hydrodynamic radius *R*_h_ were measured for a slightly ionized poly(*N*-isopropylacrylamide) (PNIPA) microgel in a dilute suspension. A decrease in *R*_h_ was observed in the temperature range between 30 °C and 35 °C, corresponding to the lower critical solution temperature of PNIPA, and an increase in |*μ*| was observed in a higher temperature range between 34 °C and 37 °C. The analysis based on electrophoresis theory for spherical polyelectrolytes indicated that the net charge of the microgel decreased as the microgel was deswollen.

## 1. Introduction

Microgels are colloidal particles of crosslinked polymers swollen by a large amount of water, the size of which ranges from tens of nanometers to several microns [1]. They can reversibly change their swelling degree and, hence, particle size in response to environmental stimuli such as pH value, temperature, and ionic strength [2,3]. This stimulus sensitivity makes microgels excellent candidates for soft materials in biomedical applications such as drug delivery and biosensing [2,3,4].

Poly(*N*-isopropylacrylamide) (PNIPA) microgel is one of the most extensively investigated thermosensitive microgels [5]. Aqueous solutions of PNIPA show a lower critical solution temperature (LCST) of 32 °C. The PNIPA microgels deswell above the LCST because of the dehydration of the polymers; the radii of the PNIPA microgels decrease with temperature above the LCST. The simplest method for preparing PNIPA microgel particles is by free radical precipitation polymerization. In this method, *N*-isopropylacrylamide (NIPA) monomer and a crosslinker are dissolved in water, and the solution is heated above the LCST; then, an initiator is added [6]. The obtained PNIPA microgel shows a narrow size distribution because a precursor particle of the microgel is formed by homogeneous nucleation [7]. For cases in which ionic initiators, such as ammonium persulfate (APS) or potassium persulfate (KPS), are used, the microgels become slightly charged because of the ionized groups imparted by the initiator [7]. Electrostatic interactions in the charged microgel play a crucial role in the colloidal stabilization of the microgel suspension and may also modulate the loading of small, charged drugs by the microgel in the drug delivery system. Thus, it is important to clarify the effect of temperature on the electrostatic properties of the ionized thermosensitive microgels.

Electrophoresis is an electrokinetic phenomenon which reflects both electrostatic and hydrodynamic properties of systems. The electrophoretic behaviors of ionized thermosensitive microgels have been investigated [5,7,8,9,10,11,12]. These previous studies showed that the absolute value of electrophoretic mobility *μ* increased with the decrease in the particle radius. This result was partly explained by the increasing charge density, although Daly et al. reported that the onset temperature of the increase in |*μ*| was slightly higher than that of the decrease in the particle radius [8]. In previous studies, experimental data of *μ* as a function of temperature were compared with different theoretical models for a hard sphere with a charged surface [7], a spherical permeable polyelectrolyte [7], and a particle covered with a polyelectrolyte layer [8,9,10,11]. These models, however, failed to sufficiently represent the experimental data [5].

In previous analysis of the electrophoretic data [5,7,8,9,10,11,12], the charge of the microgel has been commonly assumed to be independent of temperature and the particle radius. The charge defined from the number of the ionic groups on the microgel network is referred to as the bare charge, which is intrinsic to the network structure. In contrast, the charge taking into account partial screening inside the particle and/or ion binding of counterions and co-ions is called net charge, or effective charge, which varies with the environment of the microgel. The electrostatic and electrokinetic properties of the microgel are described by the net charge rather than by the bare charge. Recent theoretical and experimental studies have shown that the net charge of the microgel was affected by the swelling degree of the microgel [13,14,15,16,17]. Therefore, it is plausible to reconsider the assumption of the constant charge in the analysis of the electrophoretic data of the thermosensitive microgels as a function of temperature.

In this study, *μ* and hydrodynamic radius *R*_h_ of slightly ionized PNIPA microgels in a dilute suspension were measured as a function of temperature by electrophoretic light scattering (ELS) and dynamic light scattering (DLS), respectively, and the temperature dependence of the net charge of the microgel was investigated. The electrophoretic and hydrodynamic data were interpreted, taking the temperature dependence of the microgel net charge into consideration.

## 2. Results and Discussion

**Figure 1:**
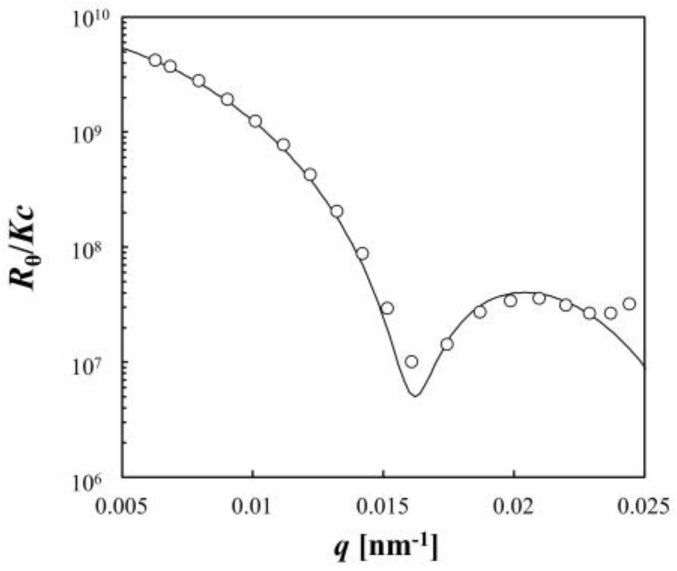
Static light scattering (SLS) data for a dilute suspension of the poly(*N*-isopropylacrylamide) (PNIPA) microgel measured at 25.0 °C and the fitted curve of the form factor for a particle with a fuzzy particle surface as described in Equations (8)–(10).

**Figure 2:**
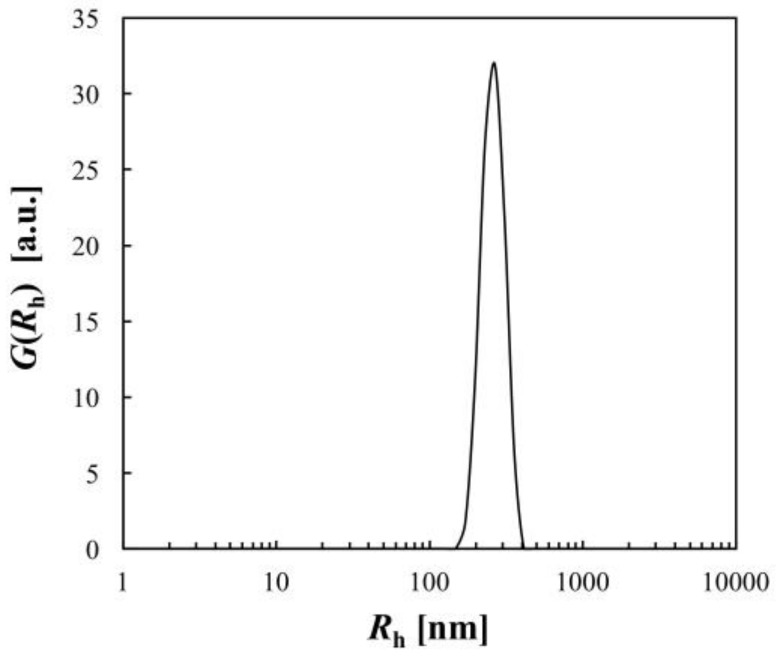
Hydrodynamic radius distribution *G*(*R*_h_) of the PNIPA microgel measured at 25.0 °C.

The characterization of the synthesized PNIPA microgel was carried out by static light scattering (SLS) and DLS measurements at 25.0 °C. The scattering curve of SLS was represented by the scattering equation for a particle with a fuzzy particle surface (Equations (8)–(10) in Materials and Methods) (Figure 1). The solid curve in Figure 1 is the fitted curve according to the equations with parameters shown in Table 1. Relatively small values of the width *σ* of the smeared particle surface and the polydispersity *σ_R_* of the particle size compared with the average particle radius <*R*> indicated that the obtained microgel was almost homogeneous and monodisperse. The distribution of *R*_h_ obtained from the DLS data (Figure 2) also represented the monodispersity of the sample. The average radius <*R*> from SLS and the average hydrodynamic radius <*R*_h_> from DLS were 2.8 × 10^2^ nm and 3.0 × 10^2^ nm, respectively, indicating good consistency with each other.

**Figure 3:**
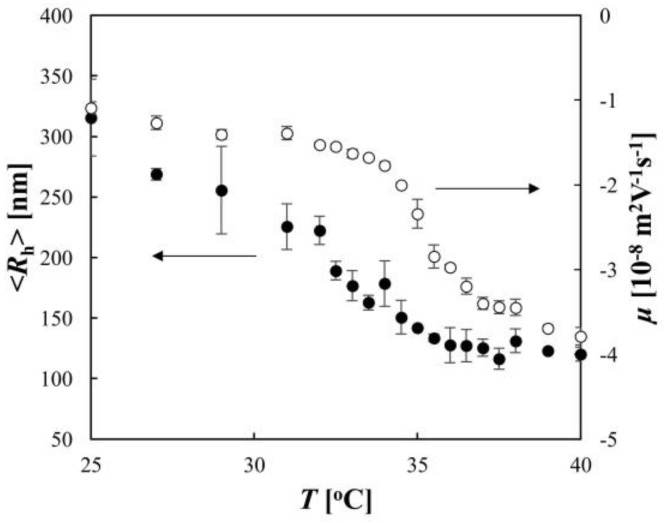
Electrophoretic mobility *μ* (open circles) and hydrodynamic radius <*R*_h_> (filled circles) as a function of temperature for the PNIPA microgel.

The thermosensitive behaviors of the PNIPA microgel were demonstrated in the DLS and ELS experiments (Figure 3). The DLS data showed that as the temperature was increased, <*R*_h_> decreased gradually at temperatures below 30 °C, and then a significant decrease in <*R*_h_> was observed in the temperature range from 30 °C to 35 °C; subsequently, <*R*_h_> decreased slightly at temperatures above 35 °C. This indicates that the noticeable deswelling of the microgel occurred in the temperature range between 30 °C and 35 °C, owing to the dehydration of PNIPA, and the midpoint of the volume change coincided with the LCST of PNIPA (32 °C). In the ELS experiment, the temperature dependence of *μ* was measured. The negative values of *μ* indicated that the microgel was negatively charged because of the sulfate group imparted by the initiator APS. The ELS data showed that as the temperature was increased, the absolute value |*μ*| of the electrophoretic mobility increased slightly at temperatures below 34 °C, and then a significant increase in |*μ*| was observed in the temperature range from 34 °C to 37 °C; subsequently, |*μ*| increased gradually at temperatures above 37 °C. The onset temperature of the increase in |*μ*| was 4 K higher than that of the decrease in <*R*_h_>, which supports the findings of a previous study [8].

In previous studies, experimental data of *μ* for ionic microgels were discussed in terms of equations based on different models [5,18,19]. One of these equations is based on a simple model of a hard sphere with a charged surface, which is equivalent to the classical Smoluchowski equation [20]. The applicability of this equation is questionable since it is not plausible to assume that all the charges are located on the surface of the microgel, particularly in the highly swollen state at low temperatures [5]. In contrast, the equation derived by Hermans and Fujita is based on a spherical polyelectrolyte model [21]. In this model, charges fixed on the particle are assumed to be uniformly distributed throughout the particle volume, and the particle is porous and permeable to small ions. The polyelectrolyte model has been used for the analysis of electrophoretic data in many experimental studies [7,18,19]. More recently, a set of equations describing *μ* for solid colloidal particles covered with an ion-penetrable layer of polyelectrolytes was derived by Ohshima [22,23]. This model has been used for analyzing *μ* of microgels in recent experimental studies [8,9,10,11] because it was proposed that microgels prepared by precipitation polymerization contained a lightly crosslinked particle periphery [24], and more charges were located in the peripheral region of the microgel in analogy to persulfate-initiated emulsifier-free polystyrene latex [7]. However, the thickness of the polyelectrolyte layer in this model cannot be determined directly from the experimental data and this value has been estimated based on various assumptions in previous studies [8,10,12]. In the present study, *μ* of the PNIPA microgel was discussed in terms of the spherical polyelectrolyte model in which no assumptions on the thickness of the polyelectrolyte layer are needed, although it is difficult to conclude which model is more suitable in the present case. In contrast with polystyrene latex, the core of PNIPA microgels should be hydrophilic even at the temperature used during the synthesis, and many sulfate groups could be located in the particle interior [7]. Moreover, even though the PNIPA microgel was prepared by precipitation polymerization in the present study, SLS data showed that the obtained microgel was a relatively homogeneous particle with a thin smeared surface. Thus, we suggest that the polyelectrolyte model is adequate for analyzing *μ* of the microgel in the present case.

In the previous studies, the temperature dependence of *μ* was not well represented by the theories described above when the charge of the microgel was assumed to be constant [5,7,8,9,10,11,12]. In the present study, the net charge of the microgel was calculated as a function of temperature by comparing the data of *μ* with the Hermans–Fujita equation for the polyelectrolyte model. According to the theory of Hermans and Fujita [21], the *μ* of spherical polyelectrolytes is described as

(1) $$\mu =\frac{ze\nu_{c}}{\eta \alpha^{2}}\left[1+\frac{2}{3}{\left(\frac{\alpha }{\kappa }\right)}^{2}\frac{1+\alpha /\left(2\kappa \right)}{1+\alpha /\kappa }\right]$$

where *z*, *e*, and *ν*_c_ represent the valence of the ionic group on the microgel network, the elementary electric charge, and the effective number of monomer units with charged moiety from the initiator per unit volume, respectively. The net charge *Z* is related to *ν*_c_ by *Z* = (4π/3) *ν*_c_ <*R*_h_>^3^. In Equation (1), *κ* is the Debye–Hückel parameter defined as

(2) $$\kappa =\underset{i}{\sum }{\left(\frac{\rho_{i}^{\infty }e^{2}z_{i}^{2}}{\varepsilon_{0}\varepsilon kT}\right)}^{1/2}$$

where *ρ_i_*^∞^ and *z_i_* are the bulk concentration and the valence of the *i*th species of mobile ions, respectively; *ε*_0_, *ε*, *k*, and *T* are the permittivity of a vacuum, the relative permittivity, the Boltzmann constant, and the absolute temperature, respectively. The parameter *α* in Equation (1) is defined as

(3) $$\alpha =\sqrt{\gamma /\eta }$$

where *η* is the viscosity and *γ* represents a frictional coefficient of the microgel per unit volume. Given that the microgel can be regarded as a porous medium and the frictional force is exerted by spheres of radius *b* corresponding to monomer units which are uniformly distributed in the microgel, *γ* is given by [25,26]

**Figure 4:**
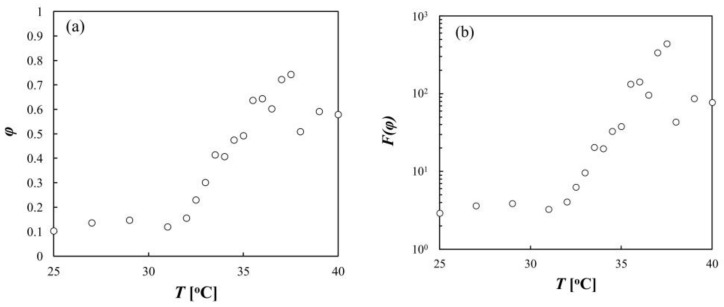
(**a**) Volume fraction φ of polymer as a function of temperature; (**b**) drag coefficient *F*(*φ*) as a function of temperature.

(4) γ=6πηbνF(φ)

where *ν* is the number density of monomers and *F*(*φ*) is a drag coefficient as a function of the volume fraction of polymer [10,26]. Koch et al. showed that *F*(*φ*) for *φ* < 0.4 and *φ* ≥ 0.4 can be represented by

(5) $$F\left(\varphi \right)=\frac{1+3{\left(\varphi /2\right)}^{2}+\left(135/64\right)\varphi \ln\varphi +16.456\varphi }{1+0.681\varphi -8.48\varphi^{2}+8.16\varphi^{3}}\;(\varphi <0.4)$$

and

(6) $$F\left(\varphi \right)=\frac{10\varphi }{{\left(1-\varphi \right)}^{3}}\;\left(\varphi \geq 0.4\right)$$

respectively [27]. From Equation (5), *F*(*φ*) = 1 as *φ* → 0. Using the relation *φ* = (4π/3) *b*^3^ *ν* and Equations (5) and (6), *φ* and *F*(*φ*) were calculated from the data of <*R*_h_> in Figure 3 and are shown as a function of temperature in Figure 4. Here, the monomer density *ν* was calculated by the relation *ν* = (*M*_w_/*M*_0_)/(4π<*R*_h_>^3^/3), where *M*_0_ is the molecular weight of the NIPA monomer (113.16). The value of *b* was estimated to be 3.4 Å from the equation *b* = (3*v*_0_*M*_0_/4π*N*_A_)^1/3^, where *N*_A_ is Avogadro’s number; as the specific volume *v*_0_ of the monomer, 0.862 cm^3^/g was used for NIPA.

**Figure 5:**
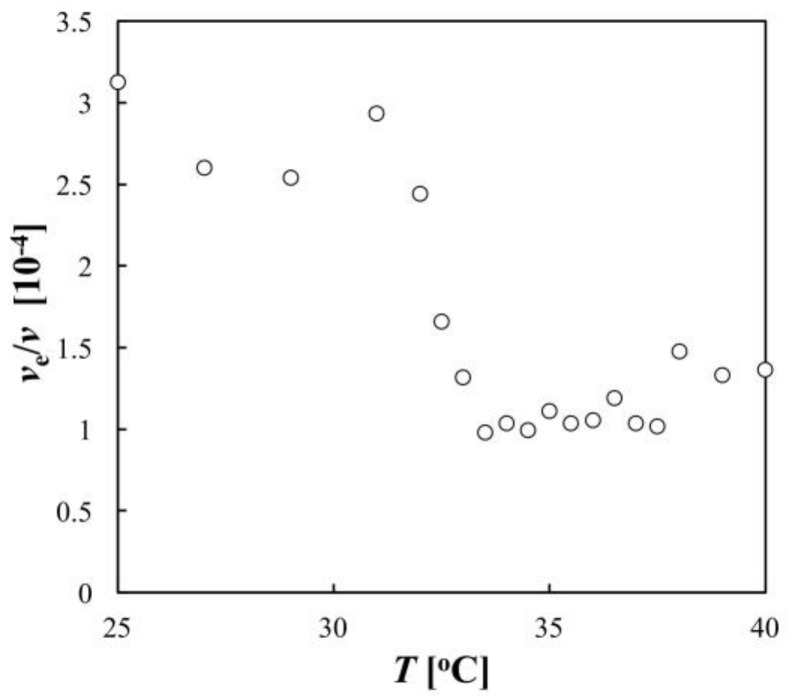
Fraction of effectively charged monomers *ν*_c_/*ν* as a function of temperature.

From the data of *μ* and <*R*_h_> in Figure 3, the fraction of effectively charged monomers *ν*_c_/*ν* was obtained by using Equations (1)–(6) (Figure 5). The value of (*ν*_c_/*ν*) reflects the net charge *Z* of the microgel because of the relation *Z* = (*ν*_c_/*ν*) (*M*_w_/*M*_0_). Figure 5 shows that as the temperature increased, *ν*_c_/*ν* was almost constant at temperatures below 31 °C, and it decreased significantly near the LCST (in the temperature range from 31 °C to 33 °C); it was then nearly constant at temperatures above 33 °C. This indicates that the net charge of the microgel decreased when the microgel deswelled. We suggest that this change in the net charge was mainly due to the change in the electrostatic property of the microgel, and the estimation of the net charge was not affected by the change in the hydrodynamic property; when *F*(*φ*) = 1 was used for all temperatures, the obtained values of *ν*_c_/*ν* were almost the same as those shown in Figure 5 (data not shown).

Figure 3 showed that the onset temperature of the increase in |*μ*| was higher than that of the decrease in <*R*_h_>. Daly et al. previously observed the same phenomenon and, on the basis of the analysis of *μ* with Ohshima’s equation, they attributed this deviation in the onsets to a three-stage deswelling process: core collapse in the first stage, partial shell collapse in the second stage, and further core collapse in the third stage [8]. However, the mechanism for this complicated deswelling process was not clarified in their study. Considering the temperature dependence of the net charge of the microgel shown in the present study, this phenomenon was explained simply as follows. In the present experimental condition, *κ*^−1^ ~ 3 × 10^−8^ m and *α* varied between 3 × 10^9^ m and 4 × 10^10^. Therefore, *α*/*κ* was sufficiently large for the first term in the bracket in Equation (1) to be regarded as negligible relative to the second term and for an approximation of (1 + *α*/2*κ*)/(1 + *α*/*κ*) ~ 1/2 to be used. This gives the following equation:

(7) $$\mu =\frac{ze}{4\pi \eta b^{3}\kappa^{2}}\left(\frac{\nu_{c}}{\nu }\right)\varphi .$$

Here, the relation *φ* = (4π/3) *b*^3^ *ν* was used. Equation (7) shows that |*μ*| is increased by increasing *φ*, but is decreased by decreasing *ν*_c_/*ν*. The gradual change in |*μ*| with temperature in the range of 31 °C to 33 °C occurs because *φ* increased approximately threefold (Figure 4) and *ν*_c_/*ν* decreased approximately threefold (Figure 5). In contrast, |*μ*| increased significantly with temperature in the range of 33 °C to 37 °C because *φ* increased further but *ν*_c_/*ν* was almost constant. Thus, the increase in the charge density of the microgel due to increasing *φ* was cancelled out by the decrease in *ν*_c_/*ν*, or the net charge *Z* in the specific temperature range, resulting in the deviation of the onset temperature of |*μ*| from that of <*R*_h_>.

Figure 5 shows that the net charge of the microgel decreases with the decrease in the swelling degree of the microgel. The effect of the swelling degree of the microgel on its net charge is not fully understood. Theoretical studies in which the excluded volume repulsion between the microgel and the ions was explicitly taken into account showed that the net charge of the microgel increased with decreasing particle radius because the ions were expelled from the inside of the microgel in the deswollen state, which led to less efficient screening of the microgel charge [13,14]. In contrast, a more mathematically rigorous approach showed that the net charge was decreased by the deswelling of the microgel because the short-ranged association between the charged microgel and the neighboring small ions became stronger, mainly because of the increasing bare charge density of the microgel [15]. The present study and other experiments of light scattering, phase behavior, and conductivity for microgel suspensions [16,17] support the latter theoretical prediction. For PNIPA microgel in the deswollen state, the environment inside the particle should be more hydrophobic because of the dehydration of the polymers, which may affect effective permittivity around the ionic groups in the microgel. It has been reported for various biopolymers that hydrophobic groups in the vicinity of an ionic group prevent dissociation of the ionic group [28,29]. The change in the dissociation constant of the ionic group could be also attributed to the observed decrease in the net charge of the microgel at high temperatures.

## 3. Conclusions

The *μ* and <*R*_h_> of slightly charged PNIPA microgel were measured as functions of temperature. The temperature dependence of *μ* was interpreted in terms of the change in the net charge of the microgel by analysis based on the spherical polyelectrolyte model. It was shown that the net charge of the microgel decreased significantly as <*R*_h_> sharply decreased near the LCST of PNIPA. This finding is important for designing colloidal stability of microgel suspensions and drug loading efficiency in microgel-based drug delivery systems.

## 4. Materials and Methods 

### 4.1. Preparation of Microgels

The NIPA, *N*,*N*’-methylenebisacrylamide (MBA) as a crosslinker, and APS as an initiator were obtained from Wako Pure Chemical Industries, Ltd., Osaka, Japan. The NIPA was recrystallized in a toluene/hexane mixture.

In a two-necked round-bottom flask, equipped with a stirrer and a reflux condenser, NIPA (0.564 g, 4.98 mmol) and MBA (0.0213 g, 0.138 mmol) were dissolved in 40 mL distilled water under stirring. After bubbling the solution with nitrogen, the solution was heated to 70 °C under a nitrogen purge and APS (0.0271 g, 0.119 mmol) dissolved in 1.2 mL water was then added to the monomer solution. The polymerization was continued for 24 h and then cooled down to room temperature. The obtained dispersion of microgels was purified by dialysis in Milli-Q water for two weeks and diluted for further measurements as described in the following.

### 4.2. SLS, DLS, and ELS Measurements

For the SLS experiment, a dilute dispersion of the microgels in 0.1 mM NaCl at a polymer concentration of *c* = 0.98 × 10^−5^ g/cm^3^ was prepared. A laboratory-made instrument equipped with a He–Ne laser (632.8 nm wavelength) and a photomultiplier on a goniometer were used for the SLS measurements. In the measurements, the scattered intensity in the angular range from 27.5° to 135° was obtained as a function of the scattering vector length *q* = (4π*n*/*λ*) sin(*θ*/2), where *n*, *λ*, and *θ* are the refractive index of the solution, the wavelength of the incident light in a vacuum, and the scattering angle, respectively. The SLS data were analyzed on the basis of the form factor *P*(*q*) for an inhomogeneous particle with a fuzzy particle surface [30,31] as

(8) $$P\left(q\right)={\left(\frac{3\left[\sin\left(qR\right)-qR\cos\left(qR\right)\right]}{{\left(qR\right)}^{3}}e^{-{\left(q\sigma \right)}^{2}/2}\right)}^{2}$$

where *R* and *σ* are the radius of the particle and the width of the smeared particle surface, respectively. Taking into account the polydispersity of the particles, the excess Rayleigh ratio *R_θ_* measured at a scattering angle *θ* as a function of *q* was fitted by the following equation:

(9) $$\frac{R_{\theta }}{Kc}=M_{w}\overset{\infty }{\underset{0}{\int }}P\left(q,R\right)D\left(R,R,\sigma_{R}\right)dR$$

where *K* is defined as *K* = (4π^2^*n*^2^/*N*_Α_λ^4^) (d*n*/d*c*)^2^, in which (d*n*/d*c*) is the refractive index increment and was determined to be 0.182 cm^3^/g by measurements with a differential refractometer; *M*_w_ is the weight-averaged molecular weight; and *D*(*R*, <*R*>, *σ_R_*) is a Gaussian distribution function with respect to the particle radius *R* given by

(10) $$D\left(R,\langle R\rangle ,\sigma_{R}\right)=\frac{1}{\sqrt{2\pi }\sigma_{R}}\exp\left[-\frac{{\left(R-R\right)}^{2}}{2\sigma_{R}^{2}}\right]$$

where <*R*> is the average particle radius and *σ_R_* represents the polydispersity of the particle size.

For the DLS and ELS experiments, a dilute dispersion of the microgels in 0.1 mM NaCl at *c* = 2.0 × 10^−5^ g/cm^3^ was prepared. The DLS and ELS measurements were performed with a Malvern Zetasizer Nano ZS instrument with a He–Ne laser at a wavelength of 632.8 nm at a fixed scattering angle of 13°. In the DLS measurement, the z-average diffusion coefficient *D*_z_ was obtained by the cumulant analysis of the autocorrelation function, and the <*R*_h_> was calculated using the Stokes–Einstein equation:

(11) $$\langle R_{h}\rangle =\frac{kT}{{6\pi \eta D}_{z}}\;.$$

The hydrodynamic radius distribution *G*(*R*_h_) was also obtained from the autocorrelation function by CONTIN analysis. In the ELS measurements, *μ* was obtained by the phase analysis light scattering (PALS) method [32].

## Figures and Tables

**Table 1:** Static light scattering (SLS) and dynamic light scattering (DLS) results for the PNIPA microgel measured at 25.0 °C.

$M_{w}$ (10^9^ g/mol)	〈R〉 (10^2^ nm)	$\sigma_{R}$ (10^2^ nm)	σ (10^2^ nm)	$\langle R_{h}\rangle$ (10^2^ nm)
8.2	2.8	0.12	0.29	3.0
